# Analysis of General Practitioners’ Attitudes and Beliefs about Psychological Intervention and the Medicine-Psychology Relationship in Primary Care: Toward a New Comprehensive Approach to Primary Health Care

**DOI:** 10.3390/healthcare9050613

**Published:** 2021-05-19

**Authors:** Attà Negri, Claudia Zamin, Giulia Parisi, Anna Paladino, Giovanbattista Andreoli

**Affiliations:** 1 Department of Human and Social Sciences, University of Bergamo, 24129 Bergamo, Italy; anna.paladino@gmail.com (A.P.); giovanbattistaandreoli@gmail.com (G.A.); 2 Italian Society of Relationship Psychoanalysis, 20135 Milano, Italy; claudia.zamin1@gmail.com; 3 UOS Prevenzione Specifica, ATS Città Metropolitana di Milano, 20122 Milano, Italy; giuparisi@ats-milano.it

**Keywords:** biopsychosocial model, primary health care psychologist, general practitioner, multi-professional health team, comprehensive primary health care

## Abstract

The biopsychosocial paradigm is a model of care that has been proposed in order to improve the effectiveness of health care by promoting collaboration between different professions and disciplines. However, its application still faces several issues. A quantitative-qualitative survey was conducted on a sample of general practitioners (GPs) from Milan, Italy, to investigate their attitudes and beliefs regarding the role of the psychologist, the approach adopted to manage psychological diseases, and their experiences of collaboration with psychologists. The results show a partial view of the psychologist’s profession that limits the potential of integration between medicine and psychology in primary care. GPs recognized that many patients (66%) would often benefit from psychological intervention, but only in a few cases (9%) were these patients regularly referred to a psychologist. Furthermore, the referral represents an almost exclusive form of collaboration present in the opinions of GPs. Only 8% of GPs would consider the joint and integrated work of the psychologist and doctor useful within the primary health care setting. This vision of the role of psychologists among GPs represents a constraint in implementing a comprehensive primary health care approach, as advocated by the World Health Organization.

## 1. The Biopsychosocial Model in Primary Health Care 

The biopsychosocial model initially proposed by Engel [1,2], which validates the definition of health previously defined by the World Health Organization [3,4], highlights the importance of the interaction among biological, psychological, and social factors on the basis of wellbeing and illness. Although this model has unanimously been recognized as valid for decades, it is rarely implemented in daily practice. The reasons for this are numerous, but among them is the persistence of the biomedical approach to diagnosis and treatment for all types of symptoms, upon which modern medicine has developed. The biomedical model has played an extraordinary role in the diagnosis and treatment of diseases. However, its exclusive use can become a “scientific counter-productive” framework according to those who claim that in any discipline, some innovation (positive at the origin), if exacerbated, could produce opposite and negative effects [5]. This is particularly true in the field of primary health care, where the individuals’ wellbeing is intrinsically connected to cultural, social, and family processes, and not just to biological and bodily functions.

The persistence of the biomedical model as the priority model in primary care leads to a reduction in the efficiency of the care system and some negative effects. Although the biomedical model, through increasingly sophisticated diagnostic procedures, has led to important advances in the biomolecular understanding and curability of *diseases*, it has also contributed to a limited focus on the person’s *illness*, that is, on the personal and social components related to the diseases. As a result, the relationship of trust between a doctor and their patient is compromised [6]. This often promotes a specific collusive relationship between them: the patient requests to be reassured by many examinations and instrumental investigations, and the doctor is often forced to adopt a defensive medicine strategy [7,8]. Even more complex to manage within a biomedical model are cases where patients presenting with several symptoms do not receive any medical evidence or explanation, despite experiencing a significant annoyance (illness). According to the contribution of Hartman, Woutersen-Koch, and Van der Horst [9], 25–50% of symptoms experienced by patients presenting to general practitioners (GPs) are often not related to any organic disease. Symptoms that cannot be explained from a medical point of view (also known as medically unexplained symptoms, MUS) are heterogeneous, and vary in severity, intensity, duration, and in the disabling impact on a patient’s life. Data from the U.K.’s Improving Access to Psychological Therapies (IAPT) program [10] demonstrated that 20–30% of consultations in primary care concerns people with MUS. This process involves many examinations, which are often inconclusive as well as expensive, and specialists’ consultations, as indicated by a recent meta-analysis [11]. In addition, the literature highlights that when patients seek explanations about their physical disorders, they often do not declare personal psychosocial difficulties [12,13], or doctors do not recognize them easily [14]. Furthermore, even if diagnosed, only between 1 and 40% of patients with psychosocial issues receive adequate treatment in the primary care system [15]. Finally, a primary care system focused on biomedical components experiences difficulty when treating and managing patients with chronic diseases who have many emotional and psychological burdens [16], and patients with proper psychological disorders. The risk of treating these patients in an exclusively biomedical model is to propose only pharmacological treatments, thus reducing the potential for treatment, or not to recognize psychological problems, such as anxiety and depression, that are hidden behind many common somatic symptoms, such as insomnia and tachycardia.

## 2. Multidisciplinary Teamwork and Primary Health Care

GPs play a central role within the health care system because they are the first institution that patients normally access to receive a diagnosis and treatments for their disease. Moreover, GPs have a unique and privileged position among the other health care professionals because they know the biomedical, social, familiar, and personal history of the patient. For this reason, they should be allowed to read the patients’ requests and problems within a psycho-socio-relational framework [17,18,19,20], but the predominance of the biomedical model and the consequent technicalization and hyperspecialization of medicine prevents the adoption of a complex and holistic approach to treatment in many cases. Among the strategies to address these difficulties is the creation of multidisciplinary teams in primary health care that allow for the recomposition of the fracture between the mind and body, and between the health professions typical of the medical model [21,22]. In the present study, we focused our attention on the possible integration of medical and psychological professionals in the Italian primary health care setting. 

In many countries, such as the Netherlands [23], Great Britain, Portugal [24,25], and Canada [26], there is a long-standing tradition of primary care psychology. Recently, New Zealand [27] has also invested resources to help young people in a primary health care setting. In all these nations, even with their differences and some critical elements, the psychologist collaborates with the family doctor or other specialists in the field of primary care [28].

In Italy, the National Health System has guaranteed the figure of the primary care physician but not the primary care psychologist since its establishment. In recent years (at the legislative level), several attempts have been made to incentivize GPs to change their organizational model, moving from an individually conducted practice to a professional activity in a network with other colleagues. In many Italian regions—one for all the most populated region, the Lombardy [29]—several health reforms have been approved in which GPs are called to integrate their competencies permanently and systematically with those of other health care professionals, such as nurses, psychologists, educators, and specialistic doctors. This aims to offer more effective and proactive primary health care services for all citizens, especially for those suffering from chronic diseases, one of the most important challenges for Western health care systems [30,31,32]. These reforms aim to improve a people-centered health care model in order to reduce the experience of depersonalization reported by patients when they have to deal with the complexity of the health care system. The figure of the psychologist is being introduced into primary care services, although with great difficulty and resistance; in most cases, the psychologists work as consultants of the GP who refers the people presenting psychological aspects; only in a few cases is there a real integration of medical and psychological competencies, for example, by visiting patients together [33,34,35].

Building and implementing multidisciplinary teamwork is an important challenge. If engaging different medical interlocutors (generalists and/or specialists) who share the same epistemology, linguistic register, and clinical procedures is a complex process, the dialogue between doctors and psychologists is even more challenging, because they refer to different epistemologies and practices. This differentiation is based on the historical split between the mind and body that has characterized Western culture [36]. The present study focused on the possibilities and constraints of this part of multi-professional collaboration in primary care. Based upon the integrated approach to care, we sought to investigate which kind of role the psychologist can play in the primary health care setting. In particular, the aim of the survey we conducted was to explore GPs’ beliefs about (a) their social representation in the psychological profession; (b) their strategies to manage the psychosocial aspects of patients’ complaints; and (c) their eventual collaboration experience with psychologists.

## 3. Materials and Methods 

An ad hoc survey composed of 32 items (Appendix A) divided into five main areas (Table 1) was developed; the questions were formulated in order to explore GPs’ attitudes and beliefs about psychological intervention and the medicine-psychology relationship in primary care. Some questions were closed-ended (with a four-step Likert scale, or yes/no answer option), some had a multiple-choice response scheme, and some were open-ended; the survey took about 10 minutes to complete. The development of the survey questions was inspired by the literature related to the biopsychosocial model [1,2] and by some preliminary studies conducted on the social representation of the psychologist and their role within primary care [14,22,33].

The participants were randomly selected from a public list of GPs of Milan. In the first semester of 2016, researchers contacted 325 GPs by phone to present the aims of the study, and subsequently, a link to the online anonymous survey was sent by email. The recruitment contacting ended when we collected 70 complete responses of GPs, deemed sufficient for an initial descriptive study, on the online platform. None of the participants received a reward for taking part in the study. The platform used for the survey was Google Modules, and the descriptive analyses were conducted through the *Jamovi* software, version 1.6. 

To categorize the responses to the open-ended questions, we adopted a grounded theory approach [37]. In the first phase, three judges independently summarized them into as few categories as possible by grouping similar answers. In the second phase, the three judges compared their categorizations to arrive at a shared synthesis of the responses. The judges were experienced psychotherapists with specific competence in health psychology. Given the exploratory nature of the study, only descriptive analyses were conducted. 

## 4. Results

Most of the GP respondents to the survey were men (*n* = 44; 64%); the average age was 55.9 years (*SD* = 7.9; 26–49: *n* = 8; 50–59: *n* = 38; 60–68: *n* = 24); 74% of GPs (*n* = 52) had previous experience in multi-professional settings other than primary care; 44% of them (*n* = 31) worked in a medical office with other GPs; the average number of assisted patients per GP was 1378 (range, 143–2000); the number of active years as a GP was, on average, 23.9 years; and 81% of GPs (*n* = 57) had two or more specialties in medical disciplines other than general medicine.

An area of the survey concerned the GPs’ evaluation of the presence of psychological needs in their patients’ requests. An indirect index of them is given by the prescription of psychotropic drugs: most respondents stated that they often (60%) or sometimes (36%) prescribed them; the few remaining GPs declared they almost never (4%) prescribed them. Most GPs stated that they almost always (11%), often (66%) or sometimes (23%) visit patients who would benefit from psychological intervention. Confirming this, the majority of GPs affirmed that they often (61%) or sometimes (39%) met patients who complained of physical diseases which had originated or had been aggravated by psychic aspects. On the part of patients, this psychological request is explicitly expressed to the doctor in most cases: only a few GPs (17%) stated that they almost never received explicit requests of this kind from their patients.

A group of survey questions asked GPs to describe their beliefs about the work and role played by psychologists, psychotherapists, psychiatrists, and counselors. For each of these mental health professionals, GPs were requested to specify the targets and goals of intervention and the reasons for referral to them. Table 2 shows the prevalent responses as categorized by judges.

According to the majority of the respondents (95%), the intervention goals of the psychiatrist were predominantly medical, namely, the diagnosis of psychiatric syndromes (especially the most severe, such as psychosis and major depression, etc.) and their pharmacological treatment; only a few GPs (5%) attributed other types of intervention goals to the psychiatrist, such as fostering patients’ problem-solving ability, wellbeing, and individual psychic resources.

The prevalent targets of psychologists for GPs were the main psychopathological symptoms together with functional and psychosomatic symptoms; their competencies and instruments did not substantially differ from those of the medical profession: to identify the problems and their causes and to find the right standard treatment for symptom reduction and resolution (56%). For other GPs, the goal of the psychologist’s intervention was generic patient well-being (4%) or scaffolding and support for patients who have certain deficits that cannot be resolved, with which, therefore, they must live (21%). The remaining respondents (19%) attributed the goals of helping people in understanding and making sense of their symptoms and problems, or in becoming aware of their needs, experiences, agency, and resources, to the psychologist.

The GPs’ beliefs and attitudes towards the psychotherapist were very similar to those attributed to the psychologist. For the majority of GPs (56%), the psychotherapist’s competencies were correctly diagnosing disorders and their causes and applying the correct standard treatments; a small proportion (4%) attributed the function of improving people’s quality of life and self-esteem to the psychotherapist; the remaining group of GPs (27%) thought that the psychotherapist’s work aims to make people aware of both causes of suffering and their own abilities and resources.

A higher proportion of GPs (29%) attributed intervention objectives other than those traditionally attributed to the medical profession to the counselor: the intervention targets were work, existential, and relational difficulties, and their intervention goals were fostering self-awareness, different points of view, personal agency, one’s potential, and resource activation. A proportion of GPs believed that the consultant had a more technical function, i.e., providing external remedies and behavioral strategies to specific and limited problems (27%). Some of the GPs thought that the counselor had functions of scaffolding and supporting people with deficits (27%). The purely medical objectives, namely, the diagnosis and treatment of mental disorders, were attributed to the consultant by only a small proportion of the GPs (17%).

The survey also investigated the propensity of GPs to collaborate with psychologists, integrating their expertise. The majority of the respondents considered that their medical training had provided much (24%) or enough (47%) evidence for the utility of medicine and psychology integration, while for the remaining GPs, such evidence had been provided to a small degree (24%) or had not been conveyed at all (5%). The majority of GPs (66%), if they had the opportunity, would make room for a psychologist within their practice. Among these GPs, some (46%) viewed individual psychological consultations carried out by a psychologist as useful, some (11%) would conduct group sessions for patients with similar pathologies together with a psychologist, and others (8%) would conduct some visits together with a psychologist for specific patients. Only a few GPs (7%) had the opinion that the work of the GP and that of the psychologist overlapped and hindered each other, whereas for all the others, their collaboration would benefit patients’ well-being. The advantages of collaboration with a psychologist would consist of helping in the management of patients with hypochondriac and psychosomatic symptoms (47%), acceleration of the healing process of some patients (25%), help in the management of patients who show little compliance (22%), and shortening the waiting list (6%).

**Figure 1:**
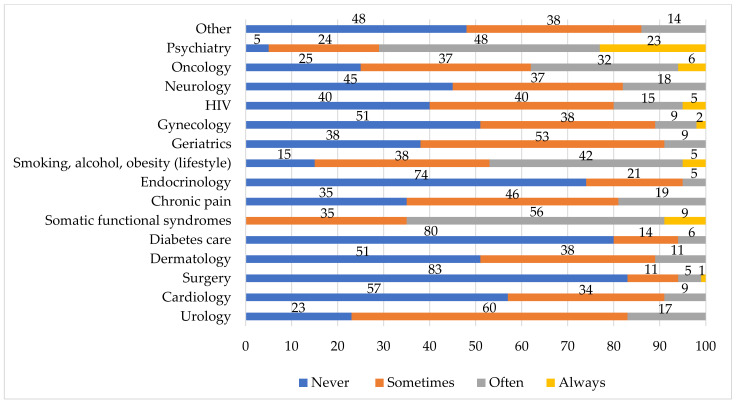
Areas of patients’ symptoms and referral frequency (%) to a psychologist.

Another area investigated by the survey regarded the actual collaboration of GPs with the psychologist in their past practice. In the offices of the majority of GPs (89%), no psychologist was present. The main form of collaboration between the GP and psychologist/psychotherapist was patient referral. A consistent proportion of GPs (42%) never referred patients to a psychologist, while the remaining GPs often (23%) or sometimes (35%) did. GPs also estimated the frequency of patient referral to a psychologist for each medical specialty (Figure 1). In most cases, a patient is only referred to a psychologist for psychiatric symptoms and functional syndromes, although it is quite surprising that, for some pathologies in which the literature recognizes a strong interconnection between psychological and physiological functioning, referral is absent for a large number of cases (cardiology = 57%; dermatology = 51%; diabetes care = 80%; chronic pain = 35%; gynecology = 51%; HIV = 40%).

The last result showed a contradiction. A large proportion of GPs stated that almost always (11%), often (66%), or sometimes (23%), they visit patients who would benefit from psychological intervention. However, only 9% of them said that they always refer patients to a psychologist when they recognize such need in the patient (and 54% said “sometimes”). In 59% of cases, in fact, the therapy of choice to respond to the psychological suffering of a patient was pharmacological treatment. Among the reasons reported for this choice are the high costs of psychological therapy (33%), the GP’s confidence in being able to help patients within the relationship with them (25%), the fear of a possible negative reaction of the patient to the proposal of psychological support (still a source of a certain amount of stigmatization), and the attempt to immediately resolve the discomfort with psychotropic drug prescriptions (15%).

## 5. Discussion

GPs are privileged observers of citizens’ diseases as well as of their health and personal histories. They deal with requests that are not only medical but often involving important and significant psychosocial needs. The survey highlighted that GPs are able to intercept heterogeneous symptoms and problems which, while showing themselves in their somatic guise, often go far beyond the organic aspect [38,39]. As results show, GPs are generally able to recognize the utility of psychological intervention for some of the problems complained of by their patients, but most of them rarely refer patients with psychological needs to a psychologist. Moreover, referring a patient to a psychologist is seen as almost the only possibility to utilize psychological competencies within primary care. Based on these data, some conclusions can be made.

Firstly, from the survey it emerged that 25% of GPs, especially those who are more experienced, believe it is possible to contain the patient’s suffering within the GP-patient relationship. This vision is derived from original Hippocratic medicine and, in our opinion, should be supported. Such unitarity, however, unfortunately lost its centrality with the affirmation of the cartesian body-mind dualism that subsequently laid the epistemic foundations for the biomedical approach. In recent decades, both the medical and psychological fields have implicitly assumed this split that favors the clinical division where the physician deals with the body and the psychologist deals with the mind [40,41]. Those who meet patients on a daily basis (GPs or psychologists) and those who listen to the suffering of patients know well how blurred the borders of this split are [42]. Certainly, a strategy to implement the application of the biopsychosocial approach in primary health care is to rethink the training career of GPs in which, at least in Italy, the holistic approach to the medical profession has lost its centrality. GPs should be equipped to read and take care of the complexities of patients, including the social, cultural, and psychological components of their wellbeing, illness, and disease [16]. Currently, to achieve these competencies in Italy, GPs need to obtain an additional specialization in psychotherapy. Here, we instead propose to equip GPs with broad skills to understand and treat the primary heath of citizens in a holistic approach. 

Secondly, the present study represents an initial reflection on the constraints and possibilities of the effective integration of medical and psychological competencies in primary health care systems. The main limitation of this study is that all GPs involved in the survey were from Milan, and thus the study detected specificity of an Italian context. In particular, the Italian primary health care system has been faced with a progressive bureaucratization of the role of the GP who, in most cases, works alone, has up to 1500 patients in their charge, and has serious time problems in managing the volume of requests addressed to them. Holistic and comprehensive care for the complexity of the patient seems unrealistic within this organizational setting, even when the GP has the best intentions and skills. For this reason, it seems increasingly necessary to evaluate the opportunity for a joint intervention of GPs and psychologists to better address these constraints and complexities. In several countries, similar studies on psychologist identities in primary care, and, more generally, on the interprofessional competencies in primary care, have been conducted [43,44], but further and more systematic investigations are needed; for example, it would be useful to conduct a multinational survey on these topics and enlarge the research scope by investigating beliefs about this new comprehensive approach to the primary health care system expressed by patients, psychologists, and other health professionals. These studies should also overcome other limitations that our research design certainly includes, such as the use of non-standardized measures, the limited number of respondents not representative of the entire population of GPs, and the fact that the data collection took place before the outbreak of the COVID-19 pandemic, which certainly may have changed the perceptions of patients and physicians with respect to the need for more comprehensive and integrated primary health care. 

Thirdly, from the GPs’ responses, it emerged that referral to mental health professionals is the privileged way to help patients from a psychological point of view. However, the ways in which the mental health professionals, and, in particular, the psychologists, can help citizens in primary care are not limited to referrals of patients with psychological symptoms by GPs. If a comprehensive and health promotion-oriented approach is adopted in primary care—scientific literature defines this approach as comprehensive primary health care (CPHC) [45,46]—then the psychologist can provide an even greater and crucial contribution. In fact, CPHC conceives health not only as the absence of disease, but as a condition to be pursued both in the absence and presence of diseases. According to this approach, primary care services should (a) be centered on the person and their relational networks; (b) adopt a life-long, promoting, preventive, and curative vision; (c) take into account the biological dimensions of the disease together with the individual (illness) and social (sickness) aspects; and (d) promote interventions aimed to enhance the social determinants of health rooted into individuals and communities. A system of CPHC services is, therefore, a proactive system capable of intercepting existing health needs, perceived or not by individuals and communities; interacting with people and communities in the places where they live, work, and interact; and developing networking skills to put into place coordinated, multi-sectoral, multi-professional, horizontal, and participatory interventions. In this type of approach, health is not a business exclusive to GPs, and psychologist’s competencies are highly beneficial, not only in terms of the treatment of patients with psychological symptoms, but also in managing the patient-health professional relationship, in analyzing the social and community components related to health, and in creating contexts that promote health in the community context, not only with a single patient. For these reasons, it is useful to consider other forms of collaboration between the GP and psychologist in addition to referral. In recent years, in some areas of Italy, forms of more comprehensive collaboration have been implemented and tested, such as the contemporary presence of a GP and psychologist during patient visits and primary health care services called “community homes” guided by some proactive, multi-professional teams [33,45,46]. However, it remains urgent to base this collaboration on a clearer and more precise conceptualization of the psychologist and the GP’s competencies and their systemic intertwining. Analysis of the responses showed that, in the GPs’ opinions, there is no clear distinction between medical and psychological intervention. In particular, the results seem to outline the psychologist’s professional figure as having the same epistemology, theories of mind, intervention aims and targets, methodology, and techniques of the other medical specialists. Psychological symptoms are viewed as sharing the same nature as organic symptoms, and so the tools to heal them are similar to other medical tools, i.e., invariant and external respect of the person’s subjectivity. On the contrary, psychological intervention does not have the direct reduction in symptoms as its primary objective, but rather the activation of the subject’s resources to re-read the experience underlying the symptoms, although this can only work with the subject’s active involvement. In this sense, the promotion of people’s resources and well-being, in sickness and health, is a core aim that the psychologist’s competencies are able to achieve [47,48]. In our survey, GPs attributed this task primarily to counselors and less to psychologists. It is beyond the scope of this work to specify the competencies of each of the mental health professionals. However, we highlight here that the survey confirmed, even for GPs, the difficulty in differentiating the competencies of these various mental health professionals and in finding a substantial dissimilarity between interventions for mental health and healing physical diseases [49,50]. This evidence leads us to question the role played over the years by psychology, which has led to the spread of a limited and incorrect conception of its areas of intervention. At least in the Italian context, the intervention of psychologists has probably not been able to propose itself within the health system, highlighting similarities and differences with other health professionals, promoting a positive and comprehensive approach to people’s health, and also applying their competencies to the health systems themselves by stimulating collaboration among the various specialists who operate within it [14,33,51]. 

In conclusion, the real integration of the GP’s and psychologist’s competencies is a crucial to offer effective primary health care to citizens and not only to manage patients with psychological disorders. The literature reports data supporting fully integrated services in primary health care because they may increase compliance, fidelity, and attendance to mental health and medical visits [52,53]. GPs constitute the first non-specialistic access point for patients’ needs, and for this, they need some basic psychological skills that can be potentiated by a joint intervention with other professionals dealing with various aspects of individuals’ health. Understanding the signs and symptoms of a disease and approaching the patient’s experience when clear and defined symptoms are present or not (in the MUS case, for example), requires GPs to be experts in mind-body relationship interconnections. By sharing significant parts of their patients’ lives and observing their evolution over the years, the GPs are able to help patients frame their illness, with consequent treatment, within a global process, reducing the fragmentation and depersonalization experience typical of modern health care systems. In this regard, it is necessary to practice medicine based on the original body-mind integration and to promote a real collaboration with all the health professionals who can favor this process. For many years, the main international health organizations have suggested following this method, promoting a comprehensive primary health care system where health is a complex goal promoted by multi-professional teams. Together with the family and community nurse (another central figure for strengthening primary care), it is also important to consider the figure of the psychologist, with the aim of taking better care of the citizens’ needs that, as even this study highlighted, are not always correctly understood and considered in their entirety. Medical and psychological sciences are, therefore, called to discuss with one another, and support one another to benefit patients’ wellbeing. It is only through the recognition of the incremental effectiveness of this collaboration that psychologists and GPs can develop creative and new solutions to promote citizens’ health and social coexistence. The COVID-19 pandemic has further highlighted the need for all primary health professionals to join forces to address the global effects of diseases [54]. Only in a creative, joint inter-professional effort can health promotion and care improve in today’s Western societies. Additionally, this not only for adult citizens but for all age groups, especially children and teenagers. A health care system in which mental health professionals are present at the primary level helps to reduce the disparities and the stigma of mental health problems and provide a much greater level of privacy to patients than when visiting specialized facilities.

## 6. Conclusions

Although the application of the biopsychosocial model has been suggested for many years to improve primary health care, the survey we conducted revealed both the potential and obstacles to this application. GPs are very sensitive to and aware of the psychological needs often present in their patients’ requests. They would also be inclined to a better response to these demands and needs. However, various obstacles prevent this propensity from taking shape in behaviors that systematically and effectively integrate medical competencies with those of mental health professionals. The present study represents an initial exploration of these obstacles and suggests the integration of GP and psychologist competencies as a way to implement a more comprehensive approach to primary health care.

## Figures and Tables

**Table 1:** Overview of the areas and questions of the survey.

Thematic Areas	Examples of Questions	Answer Options
	Degree	Year Type and year Type and year No, hospital, family counseling center, hospice, retirement home, other
Education and career information	First specialization	
	Other specialization	
	Previous medical activity in other services	
Patients’ medical conditions and need for psychological interventions	How often do you meet patients who could benefit from psychological intervention?	Never, sometimes, often, always
Beliefs and attitudes towards mental health professionals	In your opinion, what are the goals of the intervention of these different professionals: psychologist, psychotherapist, counselor and psychiatrist?	Open-ended
Attitudes towards the psychologist	If you had the opportunity, would you offer your patients a psychological intervention in your clinic?	No, yes
	In case you answered “yes” to the previous question, which psychological intervention would you offer?	Visit jointly conducted by doctor and psychologist, individual psychological/psychotherapy sessions, group posttherapy, other
	How much could the psychologist/psychotherapist’s work overlap and hinder the doctor’s work?	Not at all, a little, moderately, a lot
Features of collaborations with psychologists	How often did you refer patients to a psychologist/psychotherapist with symptoms concerning the following medical areas? (urology, cardiology, dermatology, etc.)	Never, sometimes, often, always
	As for patients you referred to a psychologist/psychotherapist, did you have feedback by the psychologist/psychotherapist? How?	No, yes; by phone, in person, other

**Table 2:** The prevalent GPs’ beliefs about mental health professionals.

Professionals	Intervention Targets	Intervention Goals	Reasons for Referral
Psychiatrist	Severe psychic symptoms (major depression, psychosis)	Diagnosis and psychopharmacological treatment	To heal and manage severe psychological symptoms
Psychologist	Psychic and functional symptoms	Diagnosis, treatment, and psychological support	To heal and manage mild psychological symptoms
Psychotherapist	Psychic and functional symptoms	Diagnosis, treatment, and psychological support	To heal and manage mild psychological symptoms
Counselor	Existential and relational difficulties	Personal skills empowerment, promotion of an active role toward their own problems	To help in managing specific life issues

## Data Availability

The data presented in this study are available on request from the corresponding author.

## Appendix A

Survey on the collaboration between GPs and psychologists

1. Do you share your clinic with other GPs?
  ○ No
  ○ Yes (How many?) _______

2. How much has your training in medicine promoted the possibility of a collaboration between medicine and psychology?
  ○ Not at all
  ○ A little
  ○ Moderately
  ○ A lot

3. In your opinion, what are the specifics of the intervention of these different professionals:
  Psychologist
  ○ Intervention targets
  ○ Intervention goals
  ○ Reasons for referral
  ○ Intervention targets
  ○ Intervention goals
  ○ Reasons for referral
  ○ Intervention targets
  ○ Intervention goals
  ○ Reasons for referral
  ○ Intervention targets
  ○ Intervention goals
  ○ Reasons for referral

4. Have you ever referred patients to a psychologist?
  ○ No
  ○ Yes

5. How often have you referred patients to a psychologist for the following medical areas?

	**Never**	**Sometimes**	**Often**	**Always**
Urology				
Cardiology				
Surgery				
Dermatology				
Diabetes care				
Somatic functional syndromes				
Chronic pain				
Endocrinology				
Smoking, alcohol, obesity (lifestyle)				
Geriatrics				
Gynecology				
HIV				
Neurology				
Oncology				
Psychiatry				
Other				

6. Regarding the patients referred, have you received any feedback from the psychologist?
  ○ No
  ○ Yes, by phone
  ○ Yes, in person
  ○ Yes, other (please specify)

7. Do you have a network of psychologists you consider “trustworthy”?
  ○ No, I do not know who to send patients to
  ○ Yes, within public facilities
  ○ Yes, within private organizations
  ○ Yes, professional psychologists (individual or associated)

8. How often do you prescribe psychotropic drugs?
  ○ Never
  ○ Sometimes
  ○ Often
  ○ Always

9. How often do you meet patients who may benefit from psychological intervention?
  ○ Never
  ○ Sometimes
  ○ Often
  ○ Always

10. How often do you refer patients who you consider may benefit from psychological intervention to a psychologist?
  ○ Never
  ○ Sometimes
  ○ Often
  ○ Always

11. For what reasons did you not propose psychological intervention to someone who would need it?
  ○ The distress was contained within the doctor-patient relationship
  ○ I opted for a pharmacological therapy
  ○ My proposal could have had a negative impact on the patient (“I’m not crazy!”)
  ○ Patient could have experienced their problem as diminished
  ○ Patient could have diminished their trust in me because I had not been able to manage the problem directly
  ○ Patient might have felt abandoned by me
  ○ I did not know who to refer them to
  ○ Patient could not economically afford a psychological consultation
  ○ Other (please specify)

12. How often do you receive requests for psychological intervention from your patients?
  ○ Never
  ○ Sometimes
  ○ Often
  ○ Always

13. How often do your patients complain about physical disorders whose origin is psychological?
  ○ Never
  ○ Sometimes
  ○ Often
  ○ Always

14. Which aspects of the relationship with patients are more difficult to manage for you?
  ○ Aggressiveness
  ○ Complaints
  ○ Mistrust
  ○ Unwarranted criticism
  ○ Other (please specify)

15. If you have the opportunity, would you offer psychological interventions to your patients in your clinic?
  ○ No
  ○ Yes, individual psychological sessions conducted by a psychologist
  ○ Yes, visits jointly conducted by doctor and psychologist
  ○ Yes, group sessions jointly conducted by doctor and psychologist for patients with similar pathologies
  ○ Yes, other (please specify)

16. A collaboration with a psychologist:
  ○ Would help me to manage patients who show little compliance
  ○ Would help me to manage patients with hypochondriac/psychosomatic symptoms
  ○ Would shorten the waiting list
  ○ Would foster a faster recovery of some patients

17. How much could the psychologist’s work overlap or hinder the doctor’s work?
  ○ Not at all
  ○ A little
  ○ Moderately
  ○ A lot

18. Is there a psychologist in your clinic?
  ○ No
  ○ Yes

19. If a psychologist is present in your clinic, how long has the partnership lasted?
  ○ 0–5 months
  ○ 6–12 months
  ○ >12 months

20. If a psychologist is present in your clinic, how often are they present in a week?
  ○ Depends on the number of appointments arranged
  ○ Twice a week
  ○ >3 times a week

21. How did the collaboration start?
  ○ On the initiative of the psychologist
  ○ On my own initiative to reduce the costs of the clinic
  ○ On my own initiative to offer an additional service to patients
  ○ There was an explicit request from patients
  ○ Other (please specify)

22. What kind of cooperation is there between you and the psychologist in your clinic?
  ○ None, s/he is a consultant to whom patients go spontaneously without my referral
  ○ Referral for psychosomatic/functional disorders
  ○ Referral for anxiety and depression disorders
  ○ Referral for adapting to living with a chronic illness
  ○ Other (please specify)

Lastly, we ask you some information about your:

23. Response date to this survey:

24. Gender:
  ○ Male
  ○ Female

25. Year of birth:
26. Year of graduation:
27. Year of specialization (specify which one):
28. Other specialties or masters (specify which one):

29. In the past, have you worked as a doctor in other public or private organizations?
  ○ No
  ○ Yes, in a hospital
  ○ Yes, in a residence for elderly
  ○ Yes, in a family counseling center
  ○ Yes, in a hospice
  ○ Yes, other (please specify)

30. Indicate the year in which you started your practice as a general practitioner:
31. Number of patients assisted:
32. Health district to which you belong:
